# Wild Bee Assemblages and Pollination Networks of Managed Emergent Wetlands in Central New York, USA

**DOI:** 10.1002/ece3.70847

**Published:** 2025-02-05

**Authors:** Molly M. Jacobson, Michael L. Schummer, Melissa K. Fierke, Paige R. Chesshire, Donald J. Leopold

**Affiliations:** ^1^ Department of Environmental Biology SUNY College of Environmental Science and Forestry Syracuse New York USA

**Keywords:** New York, plant–pollinator networks, wetland management, wetland restoration, wild bees

## Abstract

To effectively protect wild bee pollinators and the services they provide, it is critical to gather data on their distributions, life histories, and interactions with plants among a diversity of habitat types. Wetlands are underrepresented in bee surveys, despite having a great diversity of flowering plants and known importance to hundreds of species of wildlife. In this 2‐year survey of a restored wetland complex in Central New York, over 9000 bees were collected, representing ≥ 109 species in 25 genera. We recorded 337 unique plant–pollinator associations, including those previously undocumented for the wetland obligate masked bee, *Hylaeus nelumbonis* (Robertson). Floral resources and bee genera were most diverse in August, and network analyses indicated September networks were the most connected, nested, and least modular. Floral resources also shifted towards being more native over the course of the season. Results show that emergent wetlands support diverse guilds of pollinators in the latter half of the growing season, and that wetland management can produce diverse conditions conducive to wild bee habitat.

## Graphical abstract

Wetlands are underrepresented in wild bee surveys yet we find they host a diversity of bee species and offer important late‐season floral resources. Wetland restoration and management for wildlife in the northeastern United States creates a matrix of freshwater wetland habitat that helps support the landscape's wild bee fauna.

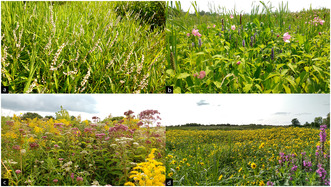

## 1 Introduction

In recent decades, declines have been documented in many wild bee species that threaten the indispensable ecological and agricultural services they provide (Colla and Packer 2008; Bartomeus et al. 2013), including an economic contribution of > $3 billion annually to food production in the U.S. (Losey and Vaughan 2006). Declines have been attributed primarily to habitat loss from agricultural expansion and intensification (Potts et al. 2010; Senapathi et al. 2015), along with exposure to insecticides and pathogens in agricultural settings (Cameron et al. 2016; Mallinger, Werts, and Gratton 2015) among other compounding factors (Goulson et al. 2015; Geslin et al. 2017). Habitat restoration and creation efforts have often focused on improving availability of floral resources in and around farm fields to offset losses in floral diversity typical of high‐intensity row crop agriculture, particularly during portions of the season when insect‐pollinated crops are not in flower (Pywell et al. 2006; M'Gonigle et al. 2015). However, substantially less attention has been paid to understanding the role of other cover types besides agriculture and grassland in supporting wild bees (but see Hanula, Ulyshen, and Horn 2016), as primary or complementary habitats (Mandelik et al. 2012).

Nontidal freshwater wetlands represent a habitat cover type that can be common in many wild and agricultural landscapes (e.g., Prairie Pothole Region of Central North America), yet remains understudied for its value to wild pollinators. In the last 250 years, > 50% of wetlands in the United States have been drained, filled, or ditched for agriculture, navigation, and urban development, with much of what remains in a degraded state (Dahl 1990). These fragmented remnant wetlands are often embedded in homogenized agricultural landscapes and may serve as refuges for rare species and sites of supplemental floral resources for diet generalist bees that could offer spillover benefits for farmers growing insect‐pollinated crops (Evans et al. 2018; Heneberg, Bogusch, and Řezác 2018; Vickruck et al. 2019; Begosh et al. 2020). In the Northeast, several diet specialist bees are associated with marsh, swamp, and bog plants like willows (*Salix* spp.), dogwoods (*Cornus* spp.), pickerelweed ( *Pontederia cordata* L.), and wetland ericads (e.g., *Vaccinium corymbosum* L. and *V. macrocarpon* Aiton, *Lyonia ligustrina* L.) (Fowler 2016). For these species, wetlands constitute their primary foraging habitat and the continued loss and fragmentation of wetlands nationwide may threaten their long‐term persistence.

Restoration efforts made possible by the North American Wetlands Conservation Act (NAWCA) and the North American Waterfowl Management Plan (NAWMP) have returned functionality as wildlife habitat, primarily aimed at waterfowl, to millions of hectares of previously drained and farmed wetlands across North America (USFWS 2018, 2020). Wetland restoration and ongoing management are usually necessary due to the severe alterations that have been made to the hydrology of most major watersheds for agriculture, residential development, and river navigation (Lang, Ingebritsen, and Griffin 2024). The restoration process typically involves the construction of impoundments which subdivides wetland systems into smaller units bordered by berms and levees, and the installation of water control structures to allow manual manipulation of the hydrology of each impoundment (Fredrickson and Taylor 1982; Strickland et al. 2009; Gray et al. 2013). Additional topography can be introduced using heavy equipment in order to increase microhabitat diversity, improve hydrology, and excavate water storage areas (“borrow ditches”) (Fredrickson and Taylor 1982; Eckler et al. 2019; Frank Morlock, NYSDEC, pers. comm.). Management of restored emergent wetlands includes using controlled seasonal drying (“drawdowns”) and flooding of individual impoundments, which mimic historic hydrology needed to cycle nutrients, aid in controlling invasive plants, and influence plant community composition (Fredrickson and Taylor 1982; Gray et al. 2013). Water levels can be manipulated to produce a variety of early‐successional conditions that can be rotated annually depending on wildlife needs, climate patterns, and landscape context, thus sustaining diverse habitat for wetland‐dependent plants and animals (Fredrickson and Taylor 1982; Fleming et al. 2012; Gray et al. 2013). The resulting emergent wetlands are dominated by herbaceous vegetation, primarily graminoids, and produce seeds, tubers, aquatic vegetation, invertebrates, and cover for high densities of migrating and breeding waterbirds and songbirds (Fredrickson and Taylor 1982; Fleming et al. 2012; Farley et al. 2022). Wetland complexes may contain dozens of impoundments and can be managed on multiple spatial scales through private–public lands partnerships to meet regional and continental conservation goals for wetland wildlife (USFWS 2018; Eckler et al. 2019).

A key component to understanding the importance of a given habitat type like wetlands to wild bees is to document species‐level associations with flowering plants and characterize them on a network level. Quantifying the structure of plant–pollinator interactions within individual systems is valuable for revealing the resource needs of pollinators, understanding ecosystem function, and identifying pollinator species critical for maintaining those functions, thus offering insight for future management recommendations. Plant–pollinator networks are complex webs of overlapping species associations (links), and the underlying topology of these interactions can reveal patterns indicative of community stability (Lance et al. 2017; Dormann 2020). Network structure can vary with time, location, and species involved (Lance et al. 2017; Endres et al. 2021) and can be measured using indices such as connectance, nestedness, or modularity. Connectance is the proportion of all possible species interactions that are realized in a network (Lance et al. 2017; Dormann 2020), and nestedness describes the degree of interaction overlap where stable, highly linked generalist species interact with other generalists as well as with poorly linked specialists (Bascompte et al. 2003; Lance et al. 2017). Network modularity can characterize the extent to which different clusters of interacting species form compartments (modules) that have few links with other distinct modules in the network (Dalsgaard et al. 2013; Lance et al. 2017). Modularity is usually directly correlated with network specialization, as generalist species tend to have more overlapping interactions across a network and not form self‐contained modules (Dormann 2020). On a species level, metrics that quantify the unique interactions of each species, such as normalized degree (ND) (Martin‐González, Dalsgaard, and Olesen 2010; Dormann 2020), can highlight which taxa are more generalized or most critical for network stability and robustness (Maia, Vaughan, and Memmott 2019). Networks that are more nested may be more resilient to species loss and disturbance, due to functional redundancy resulting from highly linked generalists stabilizing other specialized, poorly linked species in the system (Bascompte et al. 2003; Tylianakis et al. 2010; Dáttilo, Guimaraes Jr, and Izzo 2013). In less nested systems, these rare or specialized species may not be well‐buffered against the loss of their preferred interaction partners and consequently experience decreased fitness (Hoiss, Krauss, and Steffan‐Dewenter 2015). Communities with more generalists also typically experience greater connectance due to an increased number of observed interactions (Lara‐Romero et al. 2019). Overall, more connected and nested communities comprised of stable generalists as well as uncommon yet functionally important specialists (Simpson et al. 2022) can help promote ecosystems with high plant and bee diversity, may be better equipped to withstand changing climate, and can support the persistence of poorly linked bee species (Hoiss, Krauss, and Steffan‐Dewenter 2015).

Surveys to establish bee species distributions, diet and nesting preferences, and foraging phenology in underrepresented habitat types such as wetlands are critical to understanding resource needs of native bee species, assessing conservation statuses, and prescribing management actions to protect their populations and the essential services they provide (Burkle, Marlin, and Knight 2013; Goldstein and Ascher 2016; Tucker and Rehan 2016). In fact, recent studies suggest wetlands of many kinds (Moroń et al. 2008; Heneberg, Bogusch, and Řezác 2018; Vickruck et al. 2019), including managed freshwater emergent wetlands (Stephenson, Dowling, and Krementz 2020), can host a rich assemblage of wild bee pollinators. Yet, basic data on bees inhabiting emergent wetlands—their diversity, ecology, distribution, and interactions with flowering plants—are still largely lacking relative to other habitat types despite the prevalence and importance of wetlands in many landscapes. This dearth of information hinders the ability of land managers and agencies to make informed decisions that can protect and promote wild pollinators, including identifying priority habitats and aligning management goals for pollinators with those of other target wildlife. Thus, our goal for this paper was to provide novel data for the ecology of wild bees in restored, managed freshwater emergent wetlands in the northeastern United States, to fill current knowledge gaps and aid future wetland and pollinator management decisions. Specific objectives focused on describing the (1) bee diversity, (2) phenology of floral resources, and (3) monthly and seasonal structure of plant–bee networks at managed emergent wetlands in Central New York.

## 2 Methods

### 2.1 Study Area

**FIGURE 1:**
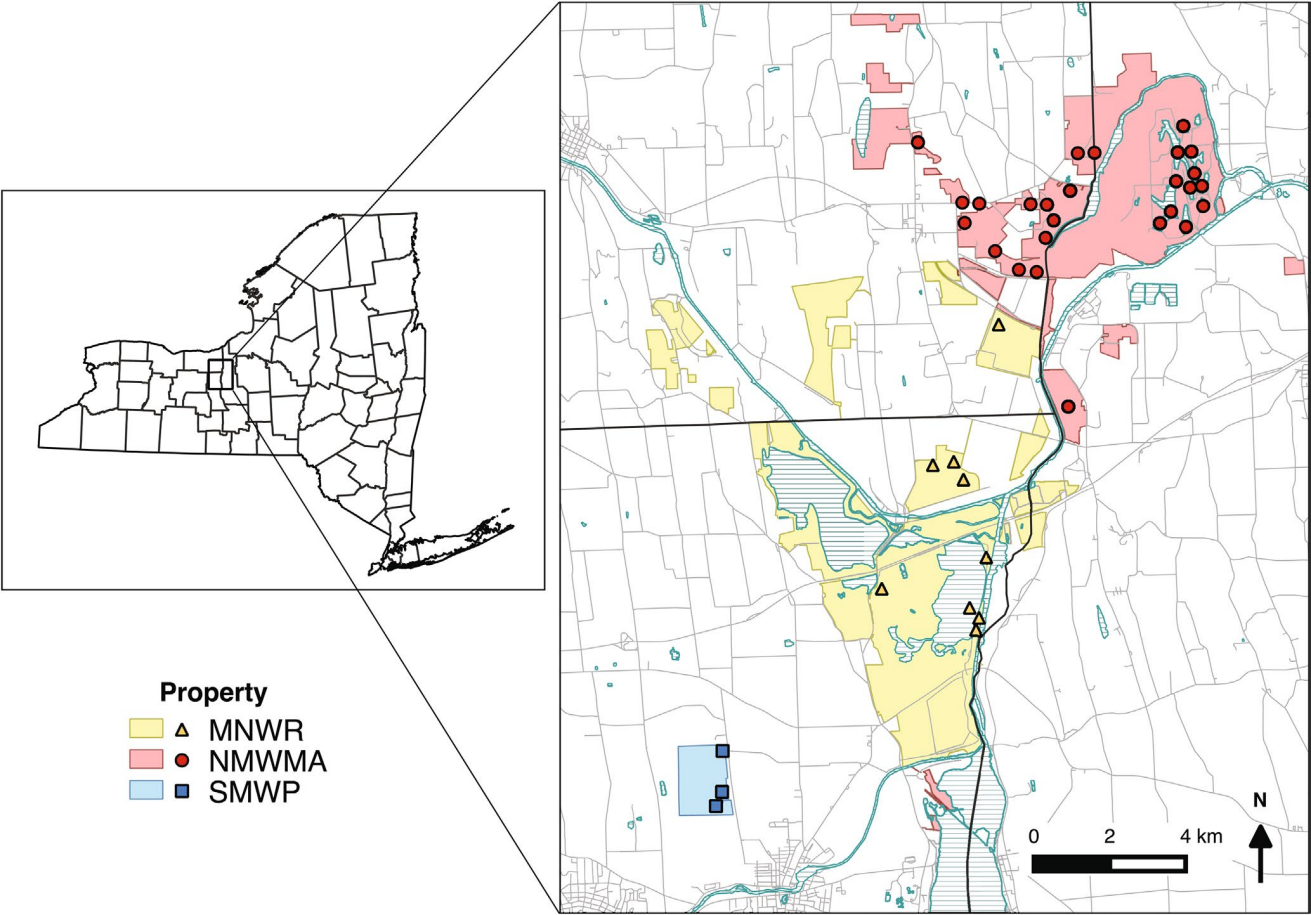
GIS map of study sites in Central New York, created using QGIS v2.4. Properties of protected lands where sampling took place are shaded. Water bodies are patterned. MNWR, Montezuma National Wildlife Refuge; NMWMA, Northern Montezuma Wildlife Management Area; SMWP, Seneca Meadows Wetlands Preserve.

This study was conducted in the Montezuma Wetlands Complex (MWC; 43.024079°N, −76.748412°W) and Seneca Meadows Wetlands Preserve (SMWP; 42.937068°N, −76.823104°W) in Central New York State, USA (Figure 1). The MWC is > 26,000 ha of wetlands and uplands with a primary focus on preservation, restoration, and management of habitats for migratory birds, particularly waterfowl (Jasikoff 2013; Eckler et al. 2019; Wagner 2020). The MWC includes the United States Fish and Wildlife Service (USFWS) Montezuma National Wildlife Refuge (MNWR; 3970 ha), New York State Department of Environmental Conservation (NYSDEC) Northern Montezuma Wildlife Management Area (NMWMA; 2860 ha), and surrounding private lands. The SMWP has 230 ha of restored or enhanced wetlands, of which 20 ha are emergent wetlands (McGraw and Larson 2019). The landscape surrounding MWC and SMWP is predominantly row crop agriculture and forest (Jasikoff 2013; Eckler et al. 2019). Historically, the region contained > 20,000 ha of contiguous wetland habitat and was subject to seasonal flooding of the Seneca River from snow melt and precipitation, but these hydrological functions that once sustained wetland heterogeneity were greatly reduced by river engineering for the New York State Canal System and draining wetlands for agriculture (Jasikoff 2013). Restored wetlands at MWC and SMWP undergo passive or active hydrological management (i.e., drawdowns) and may be subject to chemical and mechanical invasive plant control, as well as mowing and disking, which mimics river scouring activities, exposes native plant seeds for germination, and promotes a diverse plant community (Fleming et al. 2012; Farley et al. 2022). Emergent wetlands at MWC and SMWP are generally dominated by cattail (*Typha* spp.), with areas of perennial diversity including other graminoids (e.g., *Sparganium* spp., *Carex* spp., *Cyperus* spp.) and entomophilous plants (e.g., *Sagittaria* spp., *Nymphaea odorata* Aiton, many Asteraceae). Exposed soil produced by drawdowns in “moist‐soil” units results in germination of annual grasses (e.g., *Echinocloa* spp., *Panicum* spp.) and forbs (e.g., *Bidens* spp., *Persicaria* spp.) during summer that then provide forage and shelter for migrating waterfowl and songbirds (Fredrickson and Taylor 1982; Farley et al. 2022).

### 2.2 Sampling Design

Wetland impoundments were systematically sampled in 2019 and 2020. A total of 38 unique sites were surveyed, with 33 sites sampled each year based on environmental conditions and accessibility due to ongoing management. Of the 38 sites, 28 were sampled both years. Impoundments were located primarily in the MWC, with 26 at NMWMA, 9 at MNWR, and 3 at SMWP (Figure 1), and ranged in size from 2 to 670 ha (mean = 39.0, median = 10.5). For those > 35 ha, a representative 9 ha subset of the area was sampled. Sites selected for sampling were emergent wetlands and were distributed across the spectrum of hydrological conditions typical of wetland complexes (Fleming et al. 2012; Farley et al. 2022). As such, sites ranged from inundated cattail marshes with relatively little seasonal change in water levels to “moist soil” wetlands that were intentionally dewatered during the growing season to expose mudflats for seed germination and produce diverse annual and perennial vegetation. Comparison of bee and plant communities across wetlands with differing hydrological management treatments are the focus of a follow‐up study that is currently in preparation, using additional vegetation and hydrology data collected alongside bee sampling.

### 2.3 Pollinator Sampling

Pollinator surveys were conducted monthly on consecutive fair‐weather days (no precipitation, winds < 16 kph, temp. > 15.6°C) from June to September, 2019 and 2020. Sampling periods occurred over approximately 2 weeks/mo: 2019 = 7 June–24 June, 7 July–16 July, 1 August–10 August, 14–15 September and 21–22 September; 2020 = 14 June–4 July, 14 July–25 July, 10 August–20 August, and 5 September–14 September. Limited preliminary sampling occurred 20 May–25 May 2019 and 5 June 2020. Sampling was not performed until flowers of wetland species were present, to minimize biased collection of bees from adjacent habitat types.

Bees were collected with pan traps (“bee bowls”; passive) and sweep nets (active). Pan traps were 163 mL Dixie plastic soufflé cups (8 cm rim diameter, 5.25 cm base diameter × 4.5 cm depth; Georgia‐Pacific, Atlanta, GA) painted with fluorescent yellow, blue, or white paint (Guerra Paint & Pigment Corp., New York, NY) secured to 1.2 m‐height fiberglass stakes. Terrain was often inundated or held standing water, and water levels within impoundments fluctuated over the course of the season, thus to maintain sampling consistency and avoid loss of traps, all traps were fixed upon stakes regardless of site hydrology. Using a hole‐punch and mesh, drainage holes were made near the rim of each cup to prevent overflow during rain events. Traps were arranged with colors alternating along 2–4 approximately linear transects based on the unique dimensions of each site. Trap distance was consistent within each site, but adjusted across sites (5–20 m apart) to ensure coverage of the heterogeneous conditions at each impoundment of varying size. A total of 30 pan traps were placed per site in 2019 and 24 in 2020, meeting or exceeding the minimum recommended effort and trap distance described in Droege et al. (2010, 2016). Areas of impenetrable woody vegetation and open water > 1 m deep were avoided for trap placement. Traps were filled three quarters of the way with a 2:1 mixture of soapy water (Dawn Ultra blue dish soap, Procter & Gamble, Cincinnati, OH) and propylene glycol (Droege et al. 2016). Pan traps were collected after ~24 h in the field. Due to logistical limitations, only sweep netting was performed in September 2019, and passive sampling effort was reduced in the second field season, as mentioned above, in order to increase sweep netting effort and record more plant–pollinator interactions.

Sweep‐netting was performed to capture taxa not commonly collected in pan traps (e.g., *Bombus* spp. and diet specialists) and document floral associations on sampling days generally between 09:00 and 14:00 h when bees were most active (Droege et al. 2016). However, flowers observed being heavily visited after 14:00 h were opportunistically swept to maximize documentation of unique plant–pollinator interactions. One flowering plant species was swept at a time, and each sweep event lasted a maximum of 15 min, often using multiple sweep passes if a plant species existed in patches throughout a site. For plant species with only a few flowers per site, a pass consisted of stationary observation and netting/hand‐capture of any pollinators at an individual plant, while for highly abundant species, a pass involved thoroughly sweeping the entire patch (or accessible portion of the patch) once before emptying net contents into ethanol. A sweep event concluded either when 15 min had elapsed or if no further pollinating insects had been observed visiting the plant species after 2 min of observation. The number of sweep events per flowering plant species was determined by how common the species was in the landscape (i.e., how often it was encountered), length of blooming period, and observed pollinator visitation. Opportunistic hand‐capture occurred only to collect a lone individual of a previously unrecorded or rare species. Flowering plant species were identified to species level using Newcomb (1977) and Schummer et al. (2012).

Monthly pan trap and sweep data were pooled per site for statistical analyses. Sweep data from upland flowers on the edges of wetland sites were included in analyses, but are discussed accordingly. The Wetland Indicator Status reported for each plant species was obtained through the New York Flora Atlas (newyork.plantatlas.usf.edu) and are categorized as follows: obligate wetland (OBL), facultative wetland (FACW), facultative (FAC), facultative upland (FACU), and upland (UPL). Some species are classified as NI (No Indicator); all of those that were found in this study are generally found in upland habitats and thus were included as upland species here.

### 2.4 Specimen Preparation

Specimens were stored in a 70% ethanol solution from time of capture until processing. Upon removal from ethanol, specimens were washed in a mixture of Dawn dish soap and warm water to remove pollen and debris. Bees were dried using a hair dryer to prevent hair‐matting and ensure even drying necessary for identification. All specimens were pinned and labeled with metadata indicating location, date, collection method, collector, and floral record (if applicable), and given unique ID numbers. Bees were identified to the lowest taxonomic level possible, usually species, using interactive keys on DiscoverLife (www.discoverlife.org) and relevant literature (Mitchell 1960, 1962; Rehan and Sheffield 2011; Sheffield et al. 2011). All specimens in genera *Lasioglossum* (Halictidae) and *Nomada* (Apidae), and select specimens of *Hylaeus* (Colletidae), *Melissodes* (Apidae), and *Andrena* (Andrenidae), were identified by Sam Droege, USGS Native Bee Inventory & Monitoring Lab, Patuxent, Maryland. After initial examination by Sam Droege, final species determinations on specimens of *Melissodes bidentis* Cockerell were made by Michael Veit (Pepperell, MA). Information on diet specialization of bees was obtained from Fowler (2016) and Fowler and Droege (2020) unless otherwise noted. Voucher specimens are housed at SUNY‐ESF and in other research collections. All data are publicly accessible on the data archiving platform FigShare.

### 2.5 Statistical Analysis

Statistical analyses were performed in R Studio 4.3.2 (R Core Team 2023). To determine how completely the bee assemblage was sampled and provide estimates of true species richness, rarefaction tests were conducted in package “CommEcol” (Melo 2019) with Chao‐1 (Chao 1987), Jackknife (Burnham and Overton 1978), and Bootstrap estimates (Efron 1979).

A phenology plot for flowering plants was created using the function *geom_line* in the “ggplot2” package (Wickham 2016), using collection, sweep, and observational data on bloom times at each site across years to aid in visualization of floral resources available to pollinators across the growing season. Occasionally UPL plants were recorded at study sites; these species are included here as they were either present in drawn‐down impoundments or located within impoundments in close proximity to wetland flowers, and thus likely acted as floral resources for pollinators in these particular systems. For species only documented flowering during a single week increment (i.e., were likely only seen at ≤ 3 sites spanning just a few days), their line in the visual was extended halfway to the increment on either side; this decision was made partially for visibility, but also because it can reasonably be assumed these species had a longer flowering period than observed and detection was low due to infrequent encounters. As such, if these species occurred on the cusp of 2 months, they were counted for both.

Data from floral sweeps were arranged in a numerical matrix of total counts per insect species for a given plant species, and plant–pollinator interaction networks were generated using the *plotweb* function in the “bipartite” package (Dormann, Gruber, and Fruend 2008). Plant sweeps where no insects were caught were excluded from all network analyses. Interaction networks were created for each month (June, July, August, September) and for all months combined (“full season”). Links in the pooled network were color‐coded by bee family to visualize interactions more effectively. *Specieslevel* and *networklevel* functions were used to calculate network indices for each month separately and for data pooled across the entire season. At the species level, ND was calculated for each plant and pollinator species, which represents the proportion of unique interactions for a single species out of the total possible partners in the network (Martin‐González, Dalsgaard, and Olesen 2010; Dormann 2020). ND can provide a quantitative metric for bee and plant species generalization. At the network level, nestedness and connectance were calculated using weighted NODF (*wNODF*) and weighted connectance (*wC*), respectively. Weighted NODF (Almeida‐Neto et al. 2008) is a revised version of nestedness based on overlap and decreasing fills in a matrix that takes species abundance into consideration and is not biased by network size (Lance et al. 2017; Minachilis et al. 2020). Weighted connectance is a version of connectance that uses Shannon's diversity index to take differences in “link weights” into consideration (van Altena, Hemerik, and de Ruiter 2016). Both metrics range on a percentage scale of 0 (no nestedness or connectance) to 100 (perfectly nested or connected). Values closer to 0 often indicate low functional redundancy within the network that could lead to loss of poorly linked species (Tylianakis et al. 2010). Modularity (*Q*) quantifies from 0 to 1 the presence of discrete modules within a network, sets of species that interact more frequently with each other than with species outside of the module (Olesen et al. 2007; Lara‐Romero et al. 2019; Dormann 2020). Networks with modularity values closer to 100 are organized into species groups where interactions are strong within but few between (Dalsgaard et al. 2013; Dormann 2020).

For each network, a null modeling approach was used to determine the statistical significance of each network level index by comparing observed patterns to those expected by chance. Furthermore, data were standardized into *z*‐scores to allow direct comparisons of indices across networks because many indices are dependent on network size and shape (Ulrich, Almeida‐Neto, and Gotelli 2009; Dormann 2020). For every observed network, 500 randomly drawn networks were created in "bipartite" using the swap.web method (Dormann, Gruber, and Fruend 2008). This method uses an algorithm adopted from Gotelli (2000) and produces model webs where unweighted connectance and marginal totals of the original matrix are kept constant, thus maintaining the same number of dimensions as the observed web (Dormann, Gruber, and Fruend 2008; Dormann 2020). The swap.web method was selected over the vaznull method, which instead creates randomized networks with different marginal totals for each species than that of the original network (Vázquez et al. 2007). Our goal was to create randomized networks with the marginal totals per species maintained while constraining unweighted connectance. Using the selected null model method, weighted connectance, weighted NODF, and modularity could be compared between observed and null networks. For each network, observed and null values for a given index were used to calculate *z*‐scores, defined as *z* = (Obs–Null_*μ*_)/Null_*σ*_ (Dormann 2020). Values less than −1.96 or greater than 1.96 represent values significantly less than or greater than what is expected by chance at *α* = 0.05.

## 3 Results

### 3.1 Wild Bee Assemblages

A total of 9046 bees representing ≥ 109 species in 25 genera and five families were collected in 2019 and 2020 (Table 1). The most common family by abundance was Halictidae (75.2%), followed by Apidae (21.2%), Colletidae (2.8%), Andrenidae (0.44%), and Megachilidae (0.29%). In 2019, 4545 bees of ≥ 78 spp. were collected, with 4501 bees of ≥ 94 spp. collected in 2020. Pan traps captured 7599 bees (84%) of ≥ 82 spp. and 1447 (16%) of ≥ 83 spp. were captured in sweeps. There were ≥ 53 bee species collected in pan traps and sweeps, while ≥ 29 species were only collected in sweeps and ≥ 29 species only in pan traps. In June, 2641 specimens of ≥ 80 spp. were collected, 3941 of ≥ 59 spp. in July, 1938 of ≥ 73 spp. in August, and 463 of ≥ 37 spp. in September. An additional 63 individuals of 12 spp. were collected during pilot sampling in May 2019 including a single *Colletes inaequalis* Say, a species not captured during other sampling periods. Excluding pilot sampling, 25 species were common to all sampling periods. There were 23 species (21.1%) represented as singletons. The five most abundant species were *Lasioglossum zonulus* (Smith; *n* = 1924, 21.3%), *Lasioglossum versatum* (Robertson; *n* = 1259, 13.9%), *Lasioglossum oceanicum* (Cockerell; *n* = 683, 7.6%), *Agapostemon virescens* (Fabricius; *n* = 654, 7.2%), and *Apis mellifera* L. (*n* = 579, 6.4%; Table 1).

**TABLE 1:** List of bee species and morphospecies collected in restored emergent wetlands in Central New York in 2019–2020, with normalized degree for each species that had floral interaction records.

	Species	Abundance	Normalized degree
Andrenidae	*Andrena* (*Callandrena* s.l.) *helianthi* (Robertson 1891)	1	0.02
	*Andrena* (*Callandrena* s.l.) *placata* Mitchell 1960	4	0.02
	*Andrena* (*Callandrena* s.l.) *simplex* Smith 1853	4	0.02
	*Andrena* (*Cnemidandrena*) *hirticincta* Provancher 1888	1	0.02
	*Andrena* (*Gonandrena*) *fragilis* Smith 1853	2	0.02
	*Andrena* (*Holandrena*) *cressonii* Robertson 1891	1	—
	*Andrena* (*Melandrena*) *commoda* Smith 1879	2	—
	*Andrena* (*Melandrena*) *pruni* Robertson 1891	1	—
	*Andrena* (*Melandrena*) *vicina* Smith 1853	4	0.03
	*Andrena* (*Plastandrena*) *crataegi* Robertson 1893	2	0.02
	*Andrena* (*Scrapteropsis*) *alleghaniensis* Viereck 1907	1	0.02
	*Andrena* (*Taeniandrena*) *wilkella* (Kirby 1802)^†^	3	0.03
	*Andrena* (*Trachandrena*) *hippotes* Robertson 1895	3	0.02
	*Andrena* (*Trachandrena*) *nuda* Robertson 1891	3	0.02
	*Andrena* (*Trachandrena*) *spiraeana* Robertson 1895	1	0.02
	*Perdita* (*Perdita*) *octomaculata* (Say 1824)	1	0.02
	*Protandrena* (*Pterosarus*) *andrenoides* (Smith 1853)	6	0.02
Apidae	*Anthophora* (*Clisodon*) *terminalis* Cresson 1869	20	0.05
	*Anthophora* (*Melea*) *bomboides* Kirby 1837	2	—
	*Apis* (*Apis*) *mellifera* Linnaeus 1758^†^	579	0.59
	*Bombus* (*Cullumanobombus*) *griseocollis* (DeGeer 1773)	166	0.24
	*Bombus* (*Cullumanobombus*) *rufocinctus* Cresson 1863	3	0.05
	*Bombus* (*Pyrobombus*) *bimaculatus* Cresson 1863	37	0.10
	*Bombus* (*Pyrobombus*) *impatiens* Cresson 1863	222	0.44
	*Bombus* (*Pyrobombus*) *vagans* Smith 1854	10	0.15
	*Bombus* (*Subterraneobombus*) *borealis* Kirby 1837	1	0.02
	*Bombus* (*Thoracobombus*) *fervidus* (Fabricius 1798)	25	0.07
	*Ceratina* (*Zadontomerus*) *calcarata* Robertson 1900	29	0.12
	*Ceratina* (*Zadontomerus*) *dupla* Say 1837	66	0.19
	*Ceratina* (*Zadontomerus*) *mikmaqi* Rehan and Sheffield 2011	16	0.12
	*Ceratina* spp.	3	0.02
	*Melissodes* (*Eumelissodes*) *agilis* Cresson 1878	23	0.02
	*Melissodes* (*Eumelissodes*) *bidentis* Cockerell 1914	7	0.02
	*Melissodes* (*Eumelissodes*) *druriellus* (Kirby 1802)	18	0.05
	*Melissodes* (*Eumelissodes*) *trinodis* Robertson 1901	90	—
	*Melissodes* (*Heliomelissodes*) *desponsus* Smith 1854	21	—
	*Melissodes* (*Melissodes*) *bimaculatus* (Lepeletier 1825)	515	0.05

	*Melissodes* spp.	28	0.03
	*Nomada articulata* Smith 1854	1	—
	*Nomada pygmaea* Cresson 1863	5	0.02
	*Nomada* bidentate grp. sp.	2	0.02
	*Peponapis* (*Peponapis*) *pruinosa* (Say 1837)	5	—
	*Triepeolus pectoralis* (Robertson 1897)	1	0.02
	*Xylocopa* (*Xylocopoides*) *virginica* (Linnaeus 1771)	24	0.14
*Colletes compactus* Cresson 1868	1	—
Colletidae	*Colletes inequalis* Say 1837	1	—
	*Colletes latitarsis* Robertson 1891	5	0.02
	*Colletes simulans* Cresson 1868	4	0.02
	*Hylaeus* (*Hylaeus*) *annulatus* (Linnaeus 1758)	4	0.02
	*Hylaeus* (*Hylaeus*) *mesillae* (Cockerell 1896)	9	0.07
	*Hylaeus* (*Prosopis*) *affinis* (Smith 1853)	25	0.08
	*Hylaeus* (*Prosopis*) *illinoisensis*/*modestus*	13	—
	*Hylaeus* (*Prosopis*) *modestus* Say 1837	13	0.05
	*Hylaeus* (*Prosopis*) *modestus* grp sp.	36	0.20
	*Hylaeus* (*Prosopis*) *nelumbonis* (Robertson 1890)	137	0.24
	*Hylaeus* sp.	9	0.08
	*Agapostemon* (*Agapostemon*) *sericeus* (Förster 1771)	43	0.12
Halictidae	*Agapostemon* (*Agapostemon*) *splendens* (Lepeletier 1841)	360	0.05
	*Agapostemon* (*Agapostemon*) *virescens* (Fabricius 1775)	654	0.07
	*Augochlora* (*Augochlora*) *pura* (Say 1837)	72	0.14
	*Augochlorella aurata* (Smith 1853)	12	0.05
	*Dufourea novaeangliae* (Robertson 1897)	93	0.02
	*Halictus* (*Nealictus*) *parallelus* Say 1837	5	—
	*Halictus* (*Odontalictus*) *ligatus* Say 1837	70	0.05
	*Halictus* (*Protohalictus*) *rubicundus* (Christ 1791)	52	0.03
	*Halictus* (*Seladonia*) *confusus* Smith 1853	16	0.07
	*Lasioglossum* (*Dialictus*) *admirandum* (Sandhouse 1924)	52	0.03
	*Lasioglossum* (*Dialictus*) *bruneri* (Crawford 1902)	1	0.02
	*Lasioglossum* (*Dialictus*) *cattellae* (Ellis 1913)	1	0.02
	*Lasioglossum* (*Dialictus*) *coeruleum* (Robertson 1893)	5	0.02
	*Lasioglossum* (*Dialictus*) *cressonii* (Robertson 1890)	53	—
	*Lasioglossum* (*Dialictus*) *ephialtum* Gibbs 2010	290	0.07
	*Lasioglossum* (*Dialictus*) *gotham* Gibbs 2011	12	0.02
	*Lasioglossum* (*Dialictus*) *hitchensi* Gibbs 2012	23	0.03
	*Lasioglossum* (*Dialictus*) *katherineae* Gibbs 2011	1	—

	*Lasioglossum* (*Dialictus*) *laevissimum* (Smith 1853)	21	0.02
	*Lasioglossum* (*Dialictus*) *leucocomus* (Lovell 1908)	272	0.05
	*Lasioglossum* (*Dialictus*) *lineatulum* (Crawford 1906)	40	0.03
	*Lasioglossum* (*Dialictus*) *nigroviride* (Graenicher 1911)	3	—
	*Lasioglossum* (*Dialictus*) *oblongum* (Lovell 1905)	279	0.07
	*Lasioglossum* (*Dialictus*) *obscurum* (Robertson 1892)	14	0.08
	*Lasioglossum* (*Dialictus*) *oceanicum* (Cockerell 1916)	683	0.17
	*Lasioglossum* (*Dialictus*) *perpunctatum* (Ellis 1913)	2	—
	*Lasioglossum* (*Dialictus*) *pilosum* (Smith 1853)	38	—
	*Lasioglossum* (*Dialictus*) *planatum* (Lovell 1905)	3	0.02
	*Lasioglossum* (*Dialictus*) *platyparium* (Robertson 1895)	1	0.02
	*Lasioglossum* (*Dialictus*) *smilacinae* (Robertson 1897)	3	—
	*Lasioglossum* (*Dialictus*) *subviridatum* (Cockerell 1938)	10	0.02
	*Lasioglossum* (*Dialictus*) *tegulare* (Robertson 1890)	2	—
	*Lasioglossum* (*Dialictus*) *versans* (Lovell 1905)	1	0.02
	*Lasioglossum* (*Dialictus*) *versatum* (Robertson 1902)	1259	0.22
	*Lasioglossum* (*Dialictus*) *viridatum* (Lovell 1905)	2	—
	*Lasioglossum (Dialictus) weemsi* (Mitchell 1960)	1	—
	*Lasioglossum* (*Dialictus*) *zephyrus* (Smith 1853)	5	—
	*Lasioglossum (Hemihalictus*) *nelumbonis* (Robertson 1890)	86	—
	*Lasioglossum* (*Hemihalictus*) *pectorale* (Smith 1853)	7	0.02
	*Lasioglossum* (*Lasioglossum*) *coriaceum* (Smith 1853)	112	0.02
	*Lasioglossum* (*Leuhalictus*) *leucozonium* (Schrank 1781)^†^	85	0.03
	*Lasioglossum* (*Leuhalictus*) *zonulus* (Smith 1848)^†^	1924	0.19
	*Lasioglossum* (*Sphecodogastra*) *quebecense* (Crawford 1907)	17	—
	*Lasioglossum* (*Sphecodogastra*) *truncatum* (Robertson 1901)	3	0.03
	*Lasioglossum* spp.	113	0.10
	*Sphecodes davisii* Robertson 1897	2	—
	*Sphecodes* sp.	1	0.02
*Heriades* (*Neotrypetes*) *carinata* Cresson 1864	2	0.03
Megachilidae	*Hoplitis* (*Alcidamea*) *pilosifrons* (Cresson 1864)	3	0.02
	*Megachile* (*Callomegachile*) *sculpturalis* Smith 1853^†^	1	0.02
	*Megachile* (*Litomegachile*) *mendica* Cresson 1878	7	0.08
	*Megachile* (*Megachile*) *montivaga* Cresson 1878	3	0.02
	*Megachile* (*Megachile*) *relativa* Cresson 1878	2	0.03
	*Megachile* (*Sayapis*) *pugnata* Say 1837	1	—
	*Megachile* (*Xanthosarus*) *latimanus* Say 1823	3	0.02
	*Osmia* (*Diceratosmia*) *conjuncta* Cresson 1864	2	—
	*Osmia* (*Melanosmia*) *atriventris* Cresson 1864	1	—
	*Osmia* (*Melanosmia*) *bucephala* Cresson 1864	1	—

*Note:* Abundance values refer to the entire study, not solely sweep‐netting collections. Species denoted with “†” are non‐native.

Of the species documented, 90 (82.5%) are known or were documented in this study to likely be diet generalists (polylectic), while 19 (17.4%) were diet specialists (oligolectic). Comparing nesting habits, 68 species (62.4%) were ground‐nesting, 34 (31.2%) were cavity‐nesting, and 7 (6.4%) were kleptoparasites, which do not build nests. Rarefaction using a Bootstrap test estimated a minimum true richness of 124 bee species for the study area; other tests offered greater estimates (first‐order Jackknife = 141, Chao‐1 = 144). This lower bound translates to a species capture rate of 87.9%, the higher bound to 75.7%.

### 3.2 Plant–Pollinator Interactions

#### 3.2.1 Flowering Phenology

**FIGURE 2:**
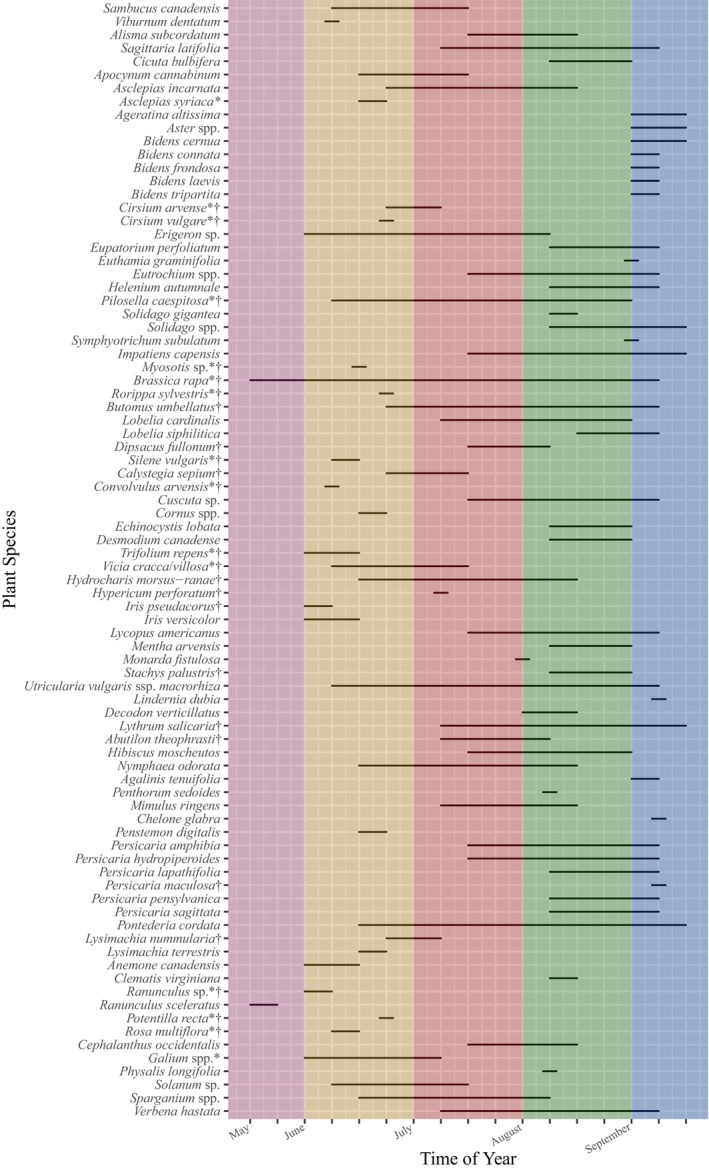
Flowering phenology of entomophilous plants recorded in restored wetlands in Central New York, 2019–2020, derived from observational data and flower sweeps, delimited by week, 20 May to 22 September. Months are delineated by color. Non‐native species are indicated by “†”, and upland species by “*”. Species only recorded from a single time point were extended half an increment on either side, and if landing on the cusp of a month were counted for both months.

**FIGURE 3:**
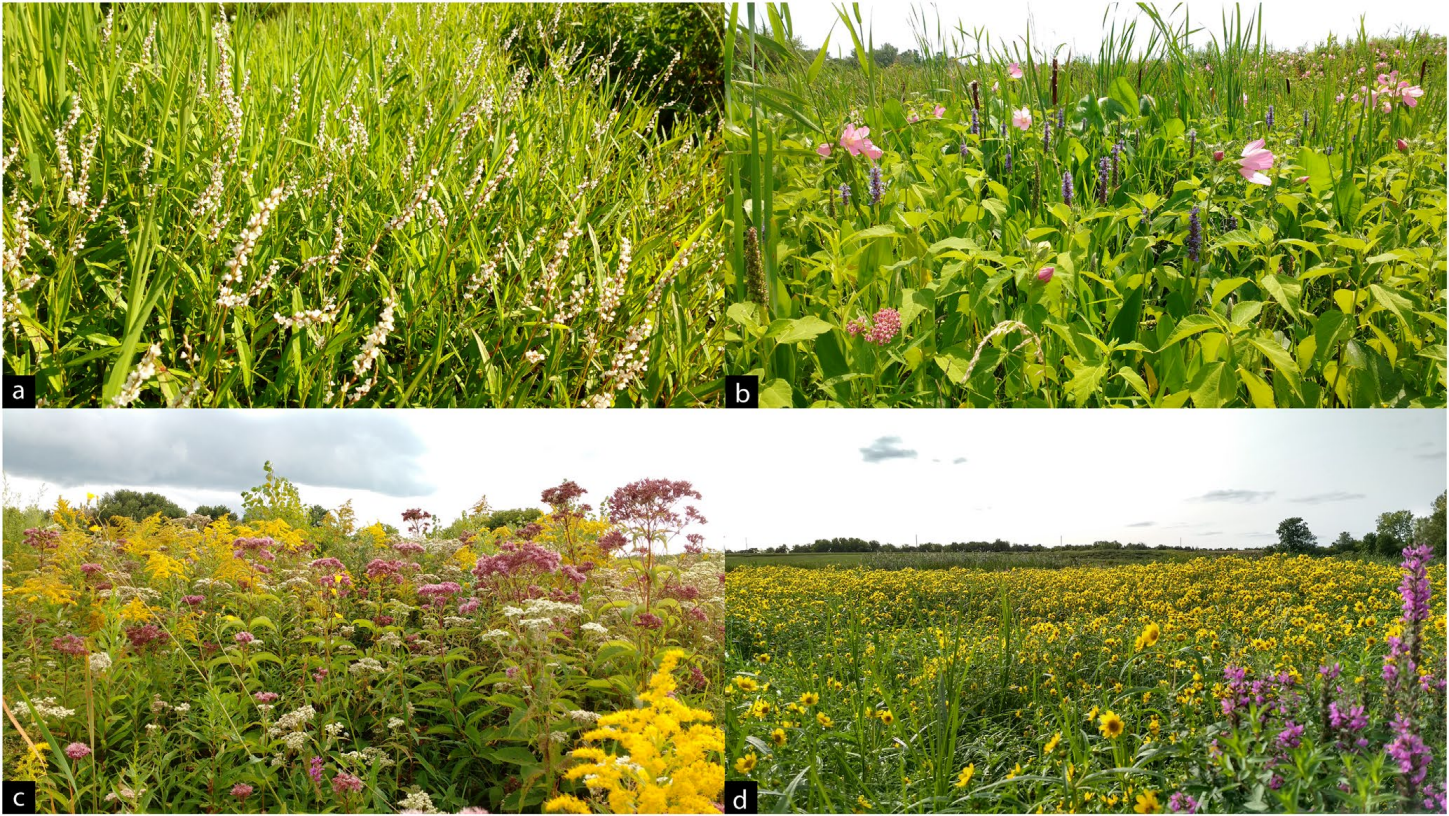
Examples of late‐summer floral resources in restored wetlands in Central New York. (a) Mat of swamp smartweed growing in shallow water. (b) Pickerelweed, swamp rose‐mallow, and swamp milkweed in shallow water. (c) Joe‐pye weeds, common boneset, and goldenrods on the wet edge of an impoundment. (d) A near‐monotypic stand of nodding bur‐marigold growing on saturated muck soil, a common sight in September. Purple loosestrife in foreground. Photographs by M. Jacobson.

Flowering period was recorded for entomophilous plants at each site, including pilot surveys in late May (Figure 2). There were two species in bloom in late May (50% were non‐native, 50% were UPL; in this case the same species—field mustard, *Brassica rapa* L.), 35 species blooming in June (51.4% non‐native, 42.9% UPL), 36 species in July (33.3% non‐native, 13.9% UPL), 46 species in August (17.4% non‐native, 4.3% UPL), and 33 species in September (9.0% non‐native, 3.0% UPL). Flowering phenologies were also broadly categorized into “early” (late May [beginning of pilot sampling]—mid‐June), “mid” (mid‐June through July), and “late” season (Aug–mid‐Sep [end of sampling]). Thus, in the early season, flowers of nine wetland species were recorded blooming, 34 species mid‐season, and 53 species late season (Figure 3). Only four species flowered from June through September: pickerelweed, bladderwort ( *Utricularia vulgaris* L. ssp. *macrorhiza* [LeConte] R.T. Clausen), field mustard, and flowering rush ( *Butomus umbellatus* L.), the latter two of which are non‐native. Field mustard was the sole species to flower continuously from May through September.

#### 3.2.2 Floral Networks

**FIGURE 4:**
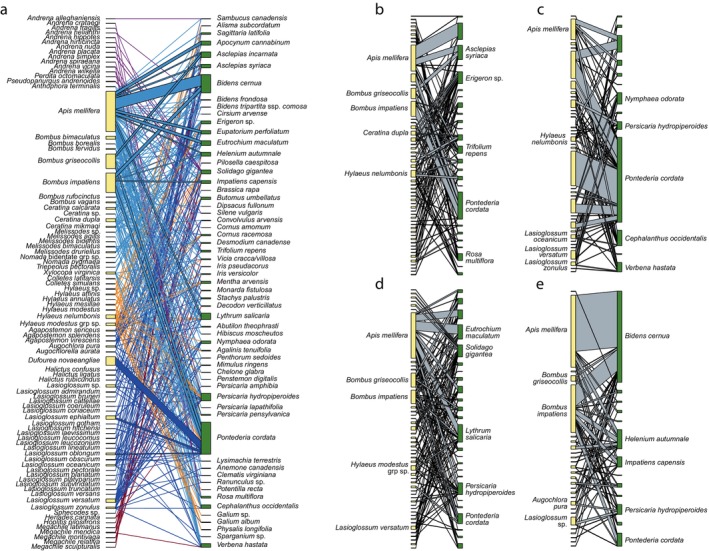
(a) Plant–pollinator interaction networks for ≥ 81 bee and 59 flowering plant species in Central New York wetlands, derived from net sweeps in 2019 and 2020. Width of interaction bars are relative to the number of individuals captured on a flower species (number of interactions). Species organized by family, interaction bars also color‐coded by bee family. (b–e) Plant–pollinator interaction networks for each sampling month, displaying the five bee taxa and five plant species with greatest normalized degree: (b) June, (c) July, (d) August, (e) September.

A total of 337 unique plant–pollinator interactions were recorded between 59 plant species (30 families) and ≥ 81 bee species across 152 sweep‐netting events (Figure 4); six additional species were swept unsuccessfully, with no insect visitors captured or observed (Table 2). By month, weighted connectance and weighted NODF were least in June (*wC* = 4.91 and *wNODF* = 6.19) and greatest in September (*wC* = 8.97 and *wNODF* = 25.14; Table 3). Weighted connectance and weighted NODF for the entire network (all months combined) was relatively low (*wC* = 4.92 and *wNODF* = 10.31, respectively). For all five networks (each individual month and all months combined), the observed values of *wC* and *wNODF* were less than what is expected by chance when compared to null models (*Z*‐test: all *p* < 0.01). Conversely, modularity by month was greatest in June (*Q* = 0.59) and least in September (*Q* = 0.26). For all five networks, the observed modularity was greater than what is expected by chance when compared to null models (*Z*‐test: all *p* < 0.01), but the difference between observed and expected modularity was much less in August (less significant) than in other months. Null model values (mean and standard deviation) for all indices are summarized in Table 3.

**TABLE 2:** Flowering plant species swept in restored wetlands in Central New York, with Wetland Indicator Status (WIS) and species‐level plant–pollinator interaction metrics. Successful sampling events were those that produced bee specimens.

Family	Flower species	Common name	WIS	Successful sampling events	Normalized degree	Mean new spp. per sweep	Mean spp. per sweep	Mean abundance per sweep
Adoxaceae	*Sambucus canadensis*	Black elderberry	FACW	1	0.03	3.0	3.0	3.0
Alismataceae	*Alisma subcordatum*	Water plantain	OBL	2	0.02	1.0	1.0	1.5
	*Sagittaria latifolia*	Arrowhead	OBL	5	0.08	1.4	2.2	3.4
Apocynaceae	*Apocynum cannabinum*	Hemp dogbane	FAC	3	0.06	1.7	2.3	14.3
	*Asclepias incarnata*	Swamp milkweed	OBL	6	0.08	1.2	2.8	10.0
	*Asclepias syriaca*	Common milkweed	UPL	3	0.12	3.3	6.5	18.5
Asteraceae	*Bidens cernua*	Nodding bur‐marigold	OBL	12	0.16	1.2	3.3	16.3
	*Bidens frondosa*	Devil's beggarticks	FACW	3	0.05	2.0	2.0	3.0
	*Bidens tripartita* ssp. *comosa*	Three‐lobed beggarticks	FACW	1	0.03	3.0	3.0	3.0
	*Cirsium arvense* ^†^	Creeping thistle	FACU	1	0.01	1.0	1.0	1.0
	*Erigeron* sp.	Fleabane	FAC	4	0.15	3.3	4.3	7.8
	*Eupatorium perfoliatum*	Common boneset	FACW	2	0.07	3.0	3.5	18.0
	*Eutrochium maculatum*	Spotted Joe‐pye weed	OBL	3	0.07	2.0	3.0	17.0
	*Helenium autumnale*	Sneezeweed	FACW	3	0.12	3.3	4.3	13.7
	*Pilosella caespitosa* ^†^	Yellow hawkweed	NI	1	0.01	1.0	1.0	3.0
	*Solidago gigantea*	Giant goldenrod	FACW	2	0.20	8.5	10.5	20.5
Balsaminaceae	*Impatiens capensis*	Spotted jewelweed	FACW	4	0.10	2.3	3.8	7.8
Brassicaceae	*Brassica rapa* ^†^	Wild mustard	UPL	1	0.01	1.0	1.0	1.0
Butomaceae	*Butomus umbellatus* ^†^	Flowering rush	OBL	2	0.08	3.5	4.0	12.5
Caprifoliaceae	*Dipsacus fullonum* ^†^	Teasel	FACU	1	0.01	1.0	1.0	1.0
Caryophyllaceae	*Silene vulgaris* ^†^	Bladder campion	NI	1	0.02	2.0	2.0	3.0
Convolvulaceae	*Convolvulus arvensis* ^†^	Field bindweed	NI	1	0.05	4.0	4.0	6.0
	*Cuscuta* sp.	Dodder	—	0	—	—	—	—
Cornaceae	*Cornus amomum*	Silky dogwood	FACW	1	0.01	1.0	1.0	1.0
	*Cornus racemosa*	Gray dogwood	FAC	2	0.08	3.5	3.5	7.0
Cucurbitaceae	*Echinocystis lobata*	Wild cucumber	FACW	0	—	—	—	—
Fabaceae	*Desmodium canadense*	Canada tick‐trefoil	FAC	1	0.05	4.0	4.0	10.0
	*Trifolium repens* ^†^	White clover	FACU	2	0.08	3.5	3.5	9.5
	*Vicia cracca* /*villosa* ^†^	Cow/hairy vetch	NI	1	0.06	5.0	5.0	10.0
Hydrocharitaceae	*Hydrocharis morsus‐ranae* ^†^	European frogbit	OBL	0	—	—	—	—
Iridaceae	*Iris pseudacorus* ^†^	Yellow flag iris	OBL	2	0.05	2.0	2.0	2.0
	*Iris versicolor*	Blue flag iris	OBL	3	0.05	1.3	1.7	3.0
Lamiaceae	*Mentha arvensis*	Wild mint	FACW	2	0.07	3.0	3.5	11.5
	*Monarda fistulosa*	Wild bergamot	FACU	1	0.05	4.0	4.0	8.0
	*Stachys palustris* ^†^	Marsh woundwort	OBL	1	0.07	6.0	6.0	13.0
Lentibulariaceae	*Utricularia vulgaris ssp. macrorhiza*	Bladderwort	OBL	0	—	—	—	—
Lythraceae	*Decodon verticillatus*	Swamp loosestrife	OBL	1	0.06	5.0	5.0	7.0
	*Lythrum salicaria* ^†^	Purple loosestrife	OBL	6	0.14	2.0	3.5	11.3
Malvaceae	*Abutilon theophrasti* ^†^	Velvetleaf	FACU	1	0.06	5.0	5.0	9.0
	*Hibiscus moscheutos*	Swamp rose mallow	OBL	2	0.02	1.0	1.0	1.0
Nymphaeaceae	*Nymphaea odorata*	White waterlily	OBL	3	0.08	2.3	3.0	9.3
Orobanchaceae	*Agalinis tenuifolia*	Common gerardia	FACW	2	0.03	1.5	2.0	4.0
Penthoraceae	*Penthorum sedoides*	Ditch stonecrop	OBL	1	0.03	3.0	3.0	4.0
Phrymaceae	*Mimulus ringens*	Allegheny monkeyflower	OBL	2	0.07	3.0	2.0	3.0
Plantaginaceae	*Chelone glabra*	White turtlehead	OBL	1	0.03	3.0	3.0	3.0
	*Penstemon digitalis*	Foxglove beardtongue	FAC	1	0.01	1.0	1.0	1.0
Polygonaceae	*Persicaria amphibia*	Water smartweed	OBL	1	0.02	3.0	3.0	4.0
	*Persicaria hydropiperoides*	Swamp smartweed	OBL	7	0.26	3.1	4.4	11.4
	*Persicaria lapathifolia*	Pale smartweed	FACW	1	0.01	1.0	1.0	1.0
	*Persicaria pensylvanica*	Pennsylvania smartweed	FACW	2	0.03	1.5	2.0	5.0
	*Persicaria sagittata*	Arrow‐leaved tearthumb	OBL	0	—	—	—	—
Pontederiaceae	*Pontederia cordata*	Pickerelweed	OBL	13	0.33	2.2	5.6	26.3
Primulaceae	*Lysimachia nummularia* ^†^	Moneywort	FACW	0	—	—	—	—
	*Lysimachia terrestris*	Swamp candles	OBL	1	0.03	3.0	3.0	3.0
Ranunculaceae	*Anemone canadensis*	Canada anemone	FACW	3	0.03	1.0	1.5	2.5
	*Clematis virginiana*	Virgin's bower	FAC	1	0.03	3.0	3.0	3.0
	*Ranunculus* sp.	Buttercup	—	3	0.03	1.0	1.5	1.0
Rosaceae	*Potentilla recta* ^†^	Sulfur cinquefoil	NI	1	0.01	1.0	1.0	2.0
	*Rosa multiflora* ^†^	Multiflora rose	FACU	3	0.10	3.0	3.7	6.0
Rubiaceae	*Cephalanthus occidentalis*	Buttonbush	OBL	2	0.09	4.0	5.5	19.0
	*Galium album* ^†^	Hedge bedstraw	FACU	2	0.06	2.5	2.5	3.0
	*Galium* sp.	Bedstraw	—	1	0.01	1.0	1.0	1.0
Solanaceae	*Physalis longifolia*	Longleaf ground cherry	NI	1	0.01	1.0	1.0	5.0
Typhaceae	*Sparganium* sp.	Bur‐reed	OBL	1	0.01	1.0	1.0	2.0
Verbenaceae	*Verbena hastata*	Swamp vervain	FACW	7	0.16	2.0	2.9	5.6

*Note:* Species denoted with “†” are non‐native. Plants with “NI” status were treated as Upland here, as all are generally associated with upland habitats.

Abbreviations: FAC, facultative; FACU, facultative upland; FACW, facultative wetland; NI, no indicator status; OBL, obligate wetland; UPL, obligate upland.

**TABLE 3:** Network metrics for bee and flowering plant interactions documented in sweep‐netting events, for each month separately and across the full season.

	June	July	August	September	Full season
Flowering plant richness	25	13	25	13	59
Bee species richness	≥ 49	≥ 33	≥ 43	≥ 25	≥ 81
Weighted NODF (*wNODF*) observed	6.188	16.538	9.786	25.143	10.309
*wNODF* null	12.106	33.375	15.483	34.438	20.033
*wNODF* sd	1.347	3.704	1.621	3.94	1.52
*wNODF z*‐score	−4.392	−4.55	−3.515	−2.359	−6.396
*wNODF p* value	< 0.00001****	< 0.00001****	0.00022***	0.0092**	< 0.00001 ****
wConnectance (*wC*) observed	4.905	8.494	7.777	8.971	4.915
*wC* null	7.172	12.16	8.712	10.284	6.541
*wC* sd	0.491	0.71	0.562	0.538	0.301
*wC z*‐score	−4.621	−5.166	−1.676	−2.442	−5.405
*wC p* value	< 0.0001****	< 0.00001****	0.047*	0.0073**	< 0.00001****
Modularity (*Q*) observed	0.590	0.412	0.425	0.268	0.405
*Q* null	0.488	0.276	0.369	0.173	0.293
*Q* sd	0.0240	0.0230	0.0240	0.025	0.014
*Q z*‐score	4.278	6.066	2.194	3.843	7.832
*Q p* value	< 0.0001****	< 0.00001****	0.014*	< 0.00001****	< 0.00001****

*Note:* For each network metric, the mean value and standard deviation obtained from 500 null models are reported, as well as standardized *z*‐scores. A *p* value of < 0.05 was considered significant. A *p*‐value of < 0.05 was considered significant and is denoted by the "*" symbol. Significance values of *p* < 0.01 are denoted with "**", < 0.001 with "***", and < 0.0001 with "****".

At the species level, the flowering plant that interacted with the greatest proportion of unique bee species in the network (i.e., the greatest ND value) was pickerelweed (0.33), followed by swamp smartweed ( *Persicaria hydropiperoides* [Michx.] Small; 0.26), and giant goldenrod ( *Solidago gigantea* Aiton; 0.19; Table 2). Giant goldenrod added the greatest mean number of new bee species per sweep event (8.5) and had the greatest mean number of bee species per sweep event (10.5), while pickerelweed had the greatest mean abundance of bees captured per sweep event (26.3 individuals). When examining bees, *A. mellifera* had the greatest ND value (0.59), followed by *Bombus impatiens* Cresson (0.44), with *Bombus griseocollis* (DeGeer) and *Hylaeus nelumbonis* (Robertson) sharing the next greatest value (0.24).

## 4 Discussion

At least 109 species of bees were recorded from restored, managed emergent wetlands in this study, including several wetland habitat specialists and diet specialists associated with wetland plants. Certain flowering plant species, particularly pickerelweed, were especially attractive to bees. These top‐performing plant species spanned a wide range of preferred hydrological conditions from wet edge to standing water, and varied in abundance and bloom period duration; all, however, were in flower in the latter half of the growing season. Floral resources in these emergent wetlands were most diverse in late summer (August–September), coinciding with the greatest species richness of recorded specialists and greatest generic richness of bees representing numerous functional guilds. Bipartite network analyses of plant–pollinator interactions indicated that the greatest nestedness and connectance, and least modularity, occurred in the month of September. Hydrologic management of restored emergent wetlands aimed at conserving vertebrate wildlife like waterfowl and other waterbirds can also benefit a diverse native bee fauna by promoting and maintaining diverse wetland conditions that sustain important floral resources for wild bees.

### 4.1 Wild Bees in Restored Wetlands

#### 4.1.1 Bee Assemblages

Bee species richness documented in this study of restored emergent wetlands (≥ 109; Table 1) was similar to other surveys of upland habitats in the northeastern US and southeastern Canada (grasslands—124, Richards et al. 2011; blueberry fields—124, Bushmann and Drummond 2015; agroecosystems—112, Tucker and Rehan 2017b; apple orchards—104, Russo et al. 2015; forest canopy and understory—90, Urban‐Mead et al. 2021) and is intermediate among North American wetland studies (40, Zarrillo and Stoner 2019; 83, Stephenson, Dowling, and Krementz 2020; 132, Vickruck et al. 2019). Estimates of true species richness indicate species capture rate of bees in wetlands at MWC and SMWP ranged from 75.7% (upper bound) to 87.9% (lower bound), which is within a similar range of surveys in Ontario (83.7%, Richards et al. 2011) and New Hampshire (80%, Tucker and Rehan 2016).

Interestingly, a few regionally common generalists— *Augochlora pura* (Say), *Halictus ligatus* Say, and especially *Augochlorella aurata* (Smith)—were infrequent to scarce in this study (0.80%, 0.77%, and 0.13%, respectively). This discrepancy was pronounced when compared to Stephenson, Dowling, and Krementz (2020), where *A. aurata* made up 47% of individuals in their survey of managed wetlands in the lower Mississippi Alluvial Valley (LMAV). While four of five most frequently collected species in our study system were also halictids, it is unclear why the above species would not utilize wetland resources as often as those in uplands in this landscape. In particular, nesting habitat would have presumably been plentiful for *A. pura* , a forest‐associated log nester, as most sites bordered forest to some degree. Determining whether this marked difference is due to wetland avoidance or simply emblematic of local conditions would require further surveys in other wetland systems in the region.

Few parasitic bees were recorded (0.14%), notably missing were *Coelioxys* (parasitic on *Megachile*) and *Bombus* (*Psithyrus*), some species of which can be fairly common in the presence of suitable hosts. It is possible that because most bees are likely not nesting within the wetland basin, parasitic species were not frequently encountered due to distance from host nests. Moreover, nest parasites that spend much of their time near the ground seeking host nest entrances may not have been attracted to the traps, which were affixed above the vegetation or water rather than placed on the ground as is common with bee bowls. Others have also documented that pan trap height affects the bee taxa collected in surveys (e.g., Tuell and Isaacs 2009; Geroff, Gibbs, and McCravy 2014).

As has been investigated by others (reviewed in Portman, Bruninga‐Socolar, and Cariveau 2020), here pan traps favored halictid bees (e.g., *Lasioglossum* and *Agapostemon*) as well as *Melissodes*, while catching few diet specialists or large‐bodied bees. Catch rates of pan traps can be greater in areas with few floral resources (Baum and Wallen 2011; Portman, Bruninga‐Socolar, and Cariveau 2020), which may partially explain the occasional presence of bees in traps located in dense cattail marshes with few to no entomophilous plants, although for the most part sites lacking floral resources had low collection rates compared to those with abundant floral resources. Several species common in traps (e.g., *Agapostemon splendens* [Lepeletier], *Melissodes bimaculatus* [Lepeletier], *Melissodes trinodis* Robertson) were rarely or never caught in sweeps, making it difficult to ascertain their roles as flower visitors in this wetland system. These phenomena highlight a potential shortcoming of this form of sampling methodology; study wetlands were located in a matrix of open, forested, and farmed land through which it is presumed bees can move freely relative to their maximum foraging distances (Gathmann and Tscharntke 2002; Greenleaf et al. 2007; Zurbuchen et al. 2010), thus bees captured in traps could represent assemblages from multiple habitat types incidentally attracted to traps while foraging or in transit (Steffan‐Dewenter et al. 2002; Russell, Ikerd, and Droege 2005). However, it does suggest bees from the surrounding landscape are generally willing to enter areas with standing water or dense vegetation to investigate potential food sources.

#### 4.1.2 Species of Note

The majority of bee species recorded here in restored wetlands are habitat and diet generalists, similar to Stephenson et al. (2018). However, several specialists utilizing these wetlands either facultatively or obligately were documented, as well as a few species unexpected for the area (Table 1). A few notable records are discussed below.

*Hylaeus nelumbonis* is associated with wetlands but its life history and ecology are still poorly understood. Specimens were collected from wetlands widely dispersed across the MWC and SMWP representing varied hydrological conditions and plant communities, and the species was present in all sampling periods (recorded 8‐Jun–22‐Sep). Mitchell (1960) lists this species as a diet specialist on lotus (*Nelumbo* spp.) and waterlily (*Nymphaea* spp.), with few published floral records since (but see Zarrillo et al. 2016; Gibbs et al. 2017). In this survey, *H. nelumbonis* was documented on 14 plant species (Figure 4); females were captured most often on pickerelweed (*n* = 7) and arrowhead ( *Sagittaria latifolia* Willd.; *n* = 3), while males frequented hemp dogbane ( *Apocynum cannabinum* L.; *n* = 4) and blue flag iris ( *Iris versicolor* L.; *n* = 4). Females were even collected on sulphur cinquefoil ( *Potentilla recta* L.), a non‐native upland species growing on the edge of one impoundment. It was only recorded once (a female) on waterlily ( *N. odorata* Aiton) despite this plant's abundance at many study sites; in addition, the presence of the bee at numerous sites where Nymphaeaceae was absent suggest it is unlikely to be relying on this sole plant family for pollen. From these floral records it can be inferred this species probably has a broader diet than previously thought, and a more focused investigation regarding pollen collection by females would aid in better understanding the dietary preferences of this enigmatic bee. In addition, locating nests would provide valuable information about the factors underlying its connection to wetland habitats.

A regionally rare species of longhorned bee, *M. bidentis* , was collected (*n* = 7, 5♀ 2♂) from five sites at the MWC, from 15 July to 10 August. Sites were located on state and federal land. The range of this species is typically the midwestern and Central United States, with recent confirmation of its presence in Manitoba, Canada (Gibbs et al. 2023). However, a handful records are known from Tompkins Co., New York, which is located at the opposite (southern) end of Cayuga Lake from the MWC, about 65 km apart over land (LaBerge 1961; GBIF 2024). These records appear to be the only documented occurrences of this species in the Northeast until those collected here. *Melissodes bidentis* is presumed according to LaBerge (1961) and Fowler (2020) to be a diet specialist on composites such as beggarticks (*Bidens*), sunflowers (*Helianthus*), and coneflowers (*Rudbeckia*). No females in this study were collected from flowers; males were taken from swamp vervain ( *Verbena hastata* L.). While beggarticks were abundant at many sites, no sunflowers or coneflowers were recorded, and the documented flight period for this bee did not overlap with the bloom period for any beggarticks species (Figure 2). Federal and state lands surrounding sites where *M. bidentis* occurred contain a matrix of wetland and upland habitats, including over 240 ha of managed grasslands on state land that comprise part of the Finger Lakes Grassland Bird Focus Area for New York Audubon's Grassland Bird Conservation Program (Morgan and Burger 2008; Eckler et al. 2019). Most likely, this bee is using these grasslands as primary habitat, many of which have been seeded with native forbs and graminoids to enhance wildlife value (Eckler et al. 2019)—its presence seems a promising sign for the response of grassland‐dependent species to preservation of remaining grasslands in the northeast.

Additionally, *Agapostemon splendens* was collected in high numbers (*n* = 360, 4.0%), being the seventh most frequently recorded species, and was found predominantly on state land. This species is an obligate sand nester (Eickwort 1981) more frequently found in coastal regions, and was only rarely collected by Stephenson (2017). Despite the majority of soils at and surrounding MWC being poorly drained muck, silty loam, or loam, there are localized loamy fine sand and very fine sandy loam deposits in and around farm fields directly adjacent to wetlands where the bee was collected most often (NRCS 2023). It is likely these sites host large *A. splendens* nesting aggregations (Frank Morlock, NYSDEC, pers. comm.). While this species would not usually be expected in inland wetland habitats, the intersection of soils, floral resources, and habitat types on multiple scales resulted in a unique local pollinator assemblage.

Finally, we detected *Bombus fervidus* (Fabricius) and *Bombus borealis* Kirby, two species of greatest conservation need in New York (NYSDEC 2015). While *B. borealis* was only recorded from a single female visiting monkeyflower ( *Mimulus ringens* L.), *B. fervidus* was relatively abundant (*n* = 25) and was collected from all three properties and in all four sampling periods (Figure 2), making frequent use of pickerelweed as a floral resource (Figure 4). *Bombus fervidus* has declined substantially in the northeastern United States (Colla and Packer 2008; Colla et al. 2012; Jacobson et al. 2018; Richardson et al. 2019), but according to state surveys, it appears to still be fairly widespread and abundant in New York compared to most neighboring states (Ascher, Kornbluth, and Goelet 2014; Bushmann and Drummond 2015; Goldstein and Ascher 2016; Tucker and Rehan 2017a; Hardy et al. 2021; White, Schlesinger, and Howard 2022) and was recently determined to be vulnerable but stable by the New York Natural Heritage Program (White, Schlesinger, and Howard 2022). *Bombus fervidus* is associated with open upland habitats, and as such decades of continued succession back to forest cover in the Northeast may be contributing to its decline in this region (Jacobson et al. 2018; Lanterman et al. 2019; Richardson et al. 2019). Richardson et al. (2019) found *B. fervidus* occurrence to be negatively associated with wetlands on multiple spatial scales in Vermont. The landscape surrounding wetlands at MWC and SMWP is a matrix of agriculture, forest, and upland meadow, which likely offered primary nesting and foraging habitat for this species. It is valuable to document *B. fervidus* using floral resources in adjacent wetland areas, which may provide supplementary forage or fill seasonal gaps.

### 4.2 Plant–Pollinator Interactions

Pickerelweed proved to be one of the most attractive wetland plants for bees in this study and an important component of pollinator networks across the season, anchoring the network and providing resources to a great diversity of diet generalist and diet specialist bees (Figure 4, Table 2). Its substantial usage by bees was notable given it was only recorded at six sites, limited to those with standing water and fairly stable water levels. Its specialist *Dufourea novaeangliae* (Robertson) was the most frequent bee visitor, found at all three properties, and over half of the bee visitors to pickerelweed were *Bombus* spp., primarily *B. impatiens* and *B. griseocollis* . Surprisingly *A. mellifera* accounted for < 1% of visitors (Figure 4). Unlike many other flowers, pickerelweed experienced substantial pollinator visitation into the evening, prompting sweep‐netting events as late as 19:00 h. The long‐blooming period of pickerelweed (Figure 2) likely offered continuous resources to sustain bumble bees through much of the colony cycle, which could be particularly valuable where passively managed (or unmanaged remnant) wetlands exist in a species‐poor agricultural matrix with otherwise sporadic flower availability (Westphal, Steffan‐Dewenter, and Tscharntke 2009; Rundlöf et al. 2014; Vickruck et al. 2019).

Giant goldenrod was only detected at two study sites, in small quantities along wetland edges, yet it yielded 17 bee species in only two sweep events, including six specialist species, and had the greatest average species richness per sweep event (10.5 spp.; Table 2). The late‐summer blooming *Solidago* genus hosts 39 specialist bees in the eastern United States (Fowler and Droege 2020) and at least 11 specialists in the northeast (Fowler 2016), indicative of its importance for wild pollinators. Wetlands with topographical variation or those managed to have wet edges as buffers between the wetland basin and surrounding uplands may offer this added benefit of productive late‐blooming wetland Asteraceae alongside those floral resources found in the basin.

While several species of smartweed were encountered, their attractiveness to bees differed vastly, with swamp smartweed by far having the greatest diversity of species interactions (Table 2, Figure 4). A perennial smartweed, it had a low (< 0.5 m), dense mat‐forming growth habit unique among *Persicaria* species in these wetlands (Schummer et al. 2012; Figure 3). It was frequented by small bees (e.g., *Hylaeus*, *Ceratina*, *Lasioglossum*) and *A. mellifera* along with many wasps. In contrast, annual smartweeds ( *P. pensylvanica* L., *P. lapathifolia* L., *Polygonum persicaria* Gray) received far fewer recorded pollinator visits. Annual smartweeds are promoted in moist‐soil units by land managers for seed production as food for migratory waterfowl (Fredrickson and Taylor 1982; Strickland et al. 2009). However, the value of some perennial smartweeds as long‐blooming nectar sources should not be overlooked if seeking to support invertebrates, which would likely also benefit insectivorous wetland songbirds during the breeding season.

Nodding bur‐marigold was the most commonly encountered entomophilous plant in actively drawn‐down wetlands and often formed expansive near‐monotypic patches (Figure 3). However, despite a nearly equal number of sweep‐netting events (12) to pickerelweed (13; due to abundance of the former, and bloom longevity in the latter) the ND value (proportion of realized to possible interaction partners) of nodding bur‐marigold was only half that of pickerelweed, and > 85% of bee visits to nodding bur‐marigold were from *A. mellifera* and *B. impatiens* . These visitation patterns indicate it was an important food source for late‐flying social bees but did not attract the diversity of solitary bees expected for its frequency, compared to sympatric Asteraceae like giant goldenrod and sneezeweed ( *Helenium autumnale* L.) which had greater average bee richness per sweep event and added more new species per sweep event as well despite being much less common on the landscape (Figure 4, Table 2). Beggarticks species are frequently managed for in moist‐soil impoundments due to their prolific seed production which constitutes a significant food source for migrating waterfowl (Fredrickson and Taylor 1982; Strickland et al. 2009); their additional value to bees is encouraging for management to concurrently benefit pollinators alongside wildlife.

While bur‐reed (*Sparganium* spp.) is traditionally considered a wind‐pollinated graminoid, in this study it was visited infrequently by *Lasioglossum* (*Dialictus*) and augochlorine bees, as well as syrphid flies. Abundant at many sites, it had staggered flowering periods such that while a fraction of plants were in bloom others were setting seed, resulting in flowers present throughout most of the growing season (Figure 3). Native bees have been known rarely to visit anemophilous wetland plants, such as *Bombus* and *Andrena* on sedges (*Carex* spp.), and *Bombus* on rushes (*Juncus* spp.; Pojar 1973; Moisan‐Deserres et al. 2014; Saunders 2018), but bees have not previously been recorded on bur‐reed in the literature. Graminoids can be easily overlooked in sweep surveys, but some could be providing abundant supplemental pollen sources to diet generalist bees when few other flowers are available on the landscape.

#### 4.2.1 Phenology

Floral resource availability patterns suggest that northeastern emergent wetlands are especially productive in August–September, supporting a diversity of pollinators during these late‐summer months. Floral resources peaked in richness in August (Figure 2), particularly so when taking into account the proportion of non‐native or upland flowering plants in each month; over the course of the season, available floral resources steadily shifted toward predominantly native species and those typical of wetlands, thus more representative of habitats wetland managers seek to restore and maintain for waterfowl and other wildlife.

While peak bee species richness was documented in June and the greatest abundance in July, due in large part to *Lasioglossum* (*Dialictus*) and *Agapostemon virescens* , the greatest generic richness occurred in August (20 genera). Furthermore, of 19 documented diet specialists, 15 were collected in August, 8 exclusively so; if expanded to the entire “late season” (Aug–Sep) then 17 specialists were collected, 12 exclusively so. Additionally, the majority of September collections by abundance were comprised of *A. mellifera* and *B. impatiens* , demonstrating the value of these late‐blooming flowers for multiple functional guilds of pollinators.

Flowering plants shown to support the greatest diversity of bees were a mix of species with long bloom periods (e.g., pickerelweed, swamp smartweed, and swamp vervain) and those with short, late bloom periods (e.g., giant goldenrod, nodding bur‐marigold), which occurred at high and low frequencies on the landscape, and in differing hydrological conditions (Figure 3, Table 2), emphasizing the importance of diverse wetland conditions to support pollinators. Some of these may provide season‐long reliable resources for generalist bees, while others offer a pulse of abundant resources for social species and specialists in late summer. Hydrological management used to control seasonal water levels and influence resulting plant communities often differs from impoundment to impoundment and may rotate annually (Fredrickson and Taylor 1982; Eckler et al. 2019; Farley et al. 2022), creating a mosaic of microhabitats and floral resources within the foraging ranges of many bee species.

In general, the floral resources available to pollinators in these restored emergent wetlands likely act as a complement to other habitat types on the landscape, such as adjacent upland forests and early‐successional habitats as well as other wetlands. Scrub–shrub wetlands, bogs, and swamps—those with a substantial woody component—offer many resources in spring that emergent wetlands usually do not, like ericaceous shrubs (e.g., *Vaccinium*, *Lyonia*, *Gaylussacia*, *Chamaedaphne*) and willows (*Salix*), which host numerous specialist bee species (Fowler and Droege 2020). Wetland complexes, natural or managed, that provide these varied habitats when landscape conditions are suitable can maximize their contribution to the diversity of the local bee fauna.

#### 4.2.2 Floral Networks

Flowering plant species appeared to vary widely in their attractiveness to bees across taxonomy, bloom phenology, and frequency in the landscape. Many abundant species at MWC that could provide steady resources appeared underutilized by bees. Arrowhead and white waterlily had relatively low documented visitation (Figure 4, Table 2), while European frogbit ( *Hydrocharis morsus‐ranae* L.) and bladderwort, two highly abundant types of submerged aquatic vegetation (SAV) with showy flowers, had no recorded visitors. Although Central New York is outside of the current northernmost range limit of the hibiscus specialist *Ptilothrix bombiformis* (Cresson) the near‐complete lack of pollinators on swamp rose mallow ( *Hibiscus moscheutos* L.) was also unexpected given its availability at MNWR within and outside of surveyed impoundments (M. Jacobson, pers. obs.; Figure 3).

By month, bee–plant networks were the least connected and nested in June and the most connected and nested in September (Table 3). Conversely, network modularity generally decreased throughout the sampling season, with networks being the most compartmentalized in June and the least compartmentalized in September. Typically, plant–pollinator networks have some degree of nestedness, such that rare or specialized species tend to interact with more common and generalized partners, helping to provide link stability for poorly linked plants or pollinators and promote greater functional redundancy across interactions (Bascompte et al. 2003; Tylianakis et al. 2010; Nielsen and Totland 2014; Simpson et al. 2022). This increased functional redundancy in more nested and connected networks, or networks with lower modularity, may lead to systems that are better equipped to withstand fluctuating environmental conditions or species loss, by safeguarding poorly linked species through shared generalist interactions (Piazzon, Larrinaga, and Santamaría 2011; Lara‐Romero et al. 2019). Thus, it is likely that in these wetlands, the greater network nestedness and connectance at the tail end of the growing season (September) promoted more redundant bee–plant interactions and greater community stability. Given the patterns of network interactions, network structure, and phenology emphasizing the importance of these late‐season resources, wetland management for wildlife that promotes these plant communities and matrices of diverse hydrological conditions is likely also simultaneously maintaining valuable pollinator habitat.

On the other hand, within plant–pollinator interaction networks, rare or poorly linked species may also be critical for facilitating species diversity and persistence in local communities. Although these species might not drive overarching community function, they may have unique roles in a system, such as contributing to functional trait diversity or acting as the primary pollinators of certain plants if these flowers receive few other visitors (Simpson et al. 2022). The importance of these uncommon species may otherwise be overlooked when networks are only analyzed as a whole. For instance, the only link for long‐leaved ground cherry ( *Physalis longifolia* Nutt.) in the network was its specialist, *Colletes latitarsis* Robertson. While this represented a one‐to‐one interaction, other interactions in our networks demonstrated how poorly linked species may also contribute to functional redundancy, such as with beggarticks. Although *A. mellifera* comprised 58.8% of all visitors to beggarticks species, a September‐blooming genus common at the MWC, their abundance was undoubtedly due to the hives maintained within the WMA (Frank Morlock, NYSDEC, pers. comm.). Removal of hives would likely greatly alter network structure, such as reducing competition with more potential partners for flower access (Geslin et al. 2017), and for beggarticks, visits by their other rarer, poorly linked partners in the network— *B. fervidus* , *Bombus rufocinctus* Cresson, *Andrena helianthi* (Robertson)—could subsequently become more frequent and important (Figure 4). With or without *A. mellifera*, other infrequent visitors to beggarticks provide some redundancy. Interestingly, we also found that not all rare species were poorly linked. Most of the bee species with the greatest ND values in our networks are indeed common diet and habitat generalists that would be expected to exert substantial influence on interaction networks in a variety of landscapes, including *A. mellifera* , *B. impatiens* , and *B. griseocollis* . However, *Hylaeus nelumbonis* was tied with *B. griseocollis* for having the third greatest ND, meaning it interacted with more plant partners than the vast majority of other bees in the network, and thus linked together and may help maintain a diversity of species. This result was surprising, given that this bee is considered regionally scarce, a wetland habitat specialist, and in the literature, a diet specialist as well. Our networks help demonstrate the value rare bees can have in their local pollination systems, in different ways, and at a scale where they may be common and make up a component of a habitat's unique character.

Importantly, network indices can be influenced by network size and be susceptible to sampling effort (Dormann, Gruber, and Fruend 2008; Jordano 2016; Vizentin‐Bugoni et al. 2016). As network size increases, connectance may decrease due to the large number of potential partners in the system (Jordano 1987), as it becomes less likely most partners will interact, for biological, behavioral, or phenological reasons, and it also becomes difficult to capture most of these interactions during sweeps. It has also been shown that modularity is less pronounced in smaller (< 50 spp.) networks (Olesen et al. 2007; Lance et al. 2017). Observed network size is subject to the ability of researchers to comprehensively collect data from all plant species in the system—in this case, species not swept were usually avoided due to either lack of any observed insect visitors, inaccessibility, or having too few flowers at peak to effectively sweep. However, in our networks, patterns of network structure remained consistent when months with similar network size were compared. Despite a greater proportion of available floral resources having been swept in June (71.4%) than August (54.3%), which resulted in an equal number of plants in each network (25 spp.), August still displayed greater connectance and nestedness, and less modularity, than those in June. A similar pattern emerged for July and September, which also shared the same plant richness (13 spp.), but September displayed greater connectance and nestedness, and less modularity (Table 3). Thus, despite similarities in network size between June and August, and July and September, results suggest plant–pollinator interactions in the latter half of the growing season (Aug–Sep) may be relatively more redundant and secure, with more native wetland floral resources, than those earlier in the season at these managed wetlands.

Emergent wetlands are often embedded in a diverse matrix of habitat types; diet and habitat generalist bees may be opportunistically using wetland floral resources when they are available and preferable, but navigate across other parts of the landscape to meet remaining temporal, nutritional, or nesting needs. Landscape context, and viewing floral resources of wetlands in conjunction with nearby flowers located on forest edges or meadows, may provide another part of the overall picture for pollination services and habitat use by wild bees in wetlands (Steffan‐Dewenter et al. 2002; Mandelik et al. 2012). Evidence from surveys here indicate emergent wetlands perform an important role on the landscape, to host flowering plants necessary for many specialists as well as offer a diversity of native floral resources for solitary and social generalists, particularly in late summer.

## 5 Conclusion

This study yielded novel data for wild bee ecology in restored, managed emergent wetlands of Central New York, which complements surveys done in the LMAV (Stephenson, Dowling, and Krementz 2020), Connecticut tidal marshes (Zarrillo and Stoner 2019), and the Prairie Pothole Region (Vickruck et al. 2019). However, further studies are needed to fill information gaps about bee diversity and floral associations in other kinds of wetlands, and in other parts of North America. These data are critical to understanding these systems and recommending effective management of wetland habitats and of native bee pollinators, both of which continue to experience significant losses worldwide (Dahl 1990; Potts et al. 2010; Bartomeus et al. 2013). This study indicated a diversity of floral resources exist in emergent wetlands for bees, across varied hydrologies, and particularly in the latter portion of the growing season. Thus, management for waterfowl and other wetland‐dependent wildlife that promotes a diversity of growing conditions also appears to benefit wild bees, as primary habitat for some specialists and complementary habitat for generalists. When emergent wetlands, especially those dominated by native plants, are located in an agricultural landscape, they can offer substantial benefits as refuges and supplemental sources of pollen and nectar for local pollinators (Evans et al. 2018; Heneberg, Bogusch, and Řezác 2018; Vickruck et al. 2019; Begosh et al. 2020). Reclamation of wetlands from agriculture at MWC and SMWP has returned floral resources at a landscape scale that are being used by at least 109 species of bees, and hydrologic management of freshwater emergent wetlands can be another valuable tool for wild bee conservation in the northeastern United States.

## Author Contributions

**Molly M. Jacobson:** conceptualization (supporting), data curation (equal), formal analysis (equal), funding acquisition (supporting), investigation (lead), methodology (supporting), visualization (lead), writing – original draft (lead), writing – review and editing (equal). **Michael L. Schummer:** conceptualization (equal), funding acquisition (lead), methodology (equal), project administration (lead), resources (equal), supervision (lead), validation (equal), writing – review and editing (equal). **Melissa K. Fierke:** conceptualization (equal), methodology (equal), project administration (supporting), resources (equal), supervision (supporting), validation (equal), writing – review and editing (equal). **Paige R. Chesshire:** data curation (equal), formal analysis (equal), visualization (supporting), writing – original draft (supporting), writing – review and editing (equal). **Donald J. Leopold:** conceptualization (supporting), methodology (supporting), project administration (supporting), resources (supporting), supervision (supporting), writing – review and editing (supporting).

## Ethics Statement

Specimens were collected on state and federal lands with appropriate permits issued by the US Fish and Wildlife Service and New York State Department of Environmental Conservation.

## Conflicts of Interest

The authors declare no conflicts of interest.

## Data Availability

The master data for all sampling events in this study across 2019 and 2020 seasons, as well as seven csv files required for reproducing all analyses in R, are hosted on figshare.org at the following DOI: https://doi.org/10.6084/m9.figshare.26452900 (Jacobson 2024). This Figshare project is titled “Wild Bee Assemblages and Pollination Networks of Restored Emergent Wetlands in Central New York, USA.” Also included in this project is the comprehensive R code used for all analyses, as well as a readme.md file that includes descriptions of every file uploaded to Figshare.
